# Characterisation of mAb104 Antibody–Drug Conjugates Targeting a Tumour-Selective HER2 Epitope

**DOI:** 10.3390/cancers17182995

**Published:** 2025-09-13

**Authors:** Sagun Parakh, Nhi Huynh, Laura D. Osellame, Diana D. Cao, Angela Rigopoulos, Benjamin Gloria, Nancy Yanan Guo, Fiona E. Scott, Zhanqi Liu, Hui K. Gan, Andrew M. Scott

**Affiliations:** 1 Tumour Targeting Laboratory, Olivia Newton-John Cancer Research Institute, Melbourne 3084, Australia; sagun.parakh@onjcri.org.au (S.P.); nhi.huynh@onjcri.org.au (N.H.); laura.osellame@onjcri.org.au (L.D.O.); diana.cao@onjcri.org.au (D.D.C.); angela.rigopoulos@onjcri.org.au (A.R.); benjamin.gloria@onjcri.org.au (B.G.); nancy.guo@onjcri.org.au (N.Y.G.); fiona.scott@onjcri.org.au (F.E.S.); zhanqi.liu@onjcri.org.au (Z.L.);; 2 Department of Medical Oncology, Austin Health, Melbourne 3084, Australia; 3 School of Cancer Medicine, Latrobe University, Melbourne 3084, Australia; 4 Department of Medicine, University of Melbourne, Melbourne 3084, Australia; 5 Department of Molecular Imaging and Therapy, Austin Health, Melbourne 3084, Australia

**Keywords:** antibody drug conjugates, HER2, epitope, trastuzumab-resistant, osimertinib-resistant

## Abstract

Background: The novel anti-HER2 antibody 104 (mAb104) targets a unique tumour-specific epitope, lacks normal tissue binding and can internalise into tumour cells, thus supporting its development into antibody drug conjugates (ADCs). Methods: We now describe the binding properties and preclinical activity of mAb104-ADCs developed through the conjugation of mAb104 via linkers to the anti-microtubule drug maytansoinoid ematansine (DM1-SMCC; DM1), topoisomerase I inhibitor, exatecan derivative (MC-GGFG-DX8951; DX8951) or microtubule disruptor monomethyl auristatin E (MC-vc-PAB-MMAE; MMAE). Results: Mab104-ADCs demonstrate dose-dependent cytotoxicity in vitro. The safety of single-dose mAb104-DX8951 was demonstrated in vivo at doses up to 10 mg/kg. MAb104-ADCs also demonstrated potent and prolonged anti-tumour activity in a range of tumour types with variable HER2 expression. Mab104-DX8951 showed significant responses in trastuzumab-resistant HER2-positive breast cancer, low HER2-expressing cancers, as well as HER2-overexpressing cancers. Conclusion: These findings indicate the potential for tumour-specific targeting of HER2-expressing tumours with mAb104-ADCs.

## Simple Summary

The human epidermal growth factor receptor 2 (HER2) is a protein expressed on cancer cells. Alterations in HER2 due to a defect in the HER2 gene or if there are excess amounts of HER2 on the cancer cell have been shown to promote cancer growth. We have previously reported on a first-in-class, tumour-specific antibody called mAb104, which has shown anti-tumour effects in HER2-positive cancers. Antibody-drug conjugates (ADCs) are a type of anticancer drug that is composed of a highly potent anticancer drug linked to an antibody that targets a specific protein (e.g., HER2). We have developed ADCs by linking mAb104 to anticancer drugs. In this paper, we demonstrate the significant anti-tumour effects of mab104-ADCs in a variety of tumour types that express HER2. Significantly, we demonstrate anti-tumour effects in tumours previously treated with HER2-directed treatments as well as in tumours with low HER2 expression.

## 1. Introduction

Many improvements in cancer treatment in the last decade have come from the use of more selective drugs, which specifically target cancer-associated proteins [1]. Antibody-drug conjugates (ADCs) are rapidly emerging as a vital class of therapeutics in the growth armamentarium of cancer drugs. ADCs combine the specificity of monoclonal antibodies to target cancer cells directly with highly potent payloads, often resulting in significant anti-tumour efficacy with a wide therapeutic window [2]. To date, twelve ADCs have been approved in oncology, of which six have been approved since 2019, highlighting the potential and effectiveness of this class of drugs [3].

The human epidermal growth factor 2 (HER2) receptor represents a prototypical molecular abnormality that is detectable in several solid tumours and a proven target in breast cancer, gastric cancer and non-small cell lung cancer (NSCLC) [4]. Furthermore, HER2-targeting therapies have shown promising activity in various other tumour types with HER2 overexpression, including gynaecological cancers, biliary tract and bladder cancer [5]. This growing effectiveness is driving a significant increase in testing for HER2 expression across various tumour types [6]. Despite the benefit of HER2-directed therapies, dose-limiting toxicities and primary or acquired resistance remain significant challenges. In addition, given the continued tumour dependence on HER2, new strategies to target HER2 are urgently needed.

We have developed novel antibodies that target conformationally exposed epitopes on the HER2 in tumour-specific conditions, and which do not bind HER2 in normal tissues [7]. We have previously described the characteristics and therapeutic potential of our lead candidate, monoclonal antibody 104 (mAb104) [7]. In contrast to other reported tumour-specific HER2-antibodies [8,9], mAb104 has shown potent anti-tumour effects in various tumour models, tumour specificity, lack of normal tissue binding and the ability to internalise in tumour cells. These characteristics have strongly supported the further development of mAb104 as an ADC as well as a naked antibody. We have created mAb104-based ADCs by conjugating mAb104 via linkers to the anti-microtubule drug maytansoinoid emtansine (DM1-SMCC; DM1), topoisomerase I inhibitor, exatecan derivative (MC-GGFG-DX8951; DX8951) and microtubule disruptor monomethyl auristatin E (MC-vc-PAB-MMAE; MMAE). Here, we describe the preclinical characterisation and therapeutic efficacy of mAb104-based ADCs in various tumour models with differential HER2 expression levels.

## 2. Materials and Methods

### 2.1. Antibodies and Antibody-Drug Conjugates

The anti-HER2 mAb104 is a murine monoclonal IgG1 produced by ONJCRI and characterised as previously described [7]. The isotype control IgG1 has been previously described by Liu et al. as LMH-3 [10]. A series of ADCs of mAb104 and isotype control IgG1 were prepared by Levena Biopharma (San Diego, CA, USA) using linkers linking the anti-microtubule drug maytansoinoid emtansine (DM1-SMCC; DM1), or topoisomerase I inhibitor, exatecan derivative (MC-GGFG-DX8951; DX8951) or microtubule disruptor monomethyl auristatin E (MC-vc-PAB-MMAE; MMAE). Pertuzumab (Genentech, San Francisco, CA, USA), Trastuzumab and Trastuzumab-emtansine (T-DM1) (Roche, Basel, Switzerland) were used as control anti-HER2 therapeutics.

### 2.2. Cell Lines

The human tumour cell lines (breast carcinoma BT-474 and HCC1954, gastric carcinoma NCI-N87 and non-small cell lung cancer (NSCLC) NCI-H2170, NCI-H1650, NCI-H522 and NCI-H838) were obtained from American Type Culture Collection and maintained in cell culture media supplemented with 10% fetal calf serum (FCS) (CSL or Bovogen, Melbourne, Victoria, Australia). The breast cancer patient-derived sample (PDX) KCC_P_4066 was provided by the National Breast Cancer Foundation Repository of primary tumours and metastases from breast cancer patients (BROCADE, Peter MacCallum Cancer Centre, Melbourne, Australia) for research use approved by the Austin Health Human Research Ethics Committee. This primary tumour sample was treated with neoadjuvant chemotherapy, and histology was grade 2 infiltrating ductal carcinoma, estrogen receptor positive, progesterone receptor negative and HER2 positive (defined as 3+ on IHC) [ER+/PR-/HER2+].

### 2.3. Flow Cytometry (FACS)

Primary anti-HER2 antibody or anti-HER2-ADC (10 µg/mL) bound to 0.5 × 10^6^ cells following incubation for 45 min at 4 °C were probed with an anti-human or anti-mouse IgG-Alexa 488 secondary antibody (Thermo Fisher^®^, Waltham, MA, USA) for another 45 min at 4 °C. Controls included cells alone and secondary antibody alone. Data was acquired on a BD FACS Canto II (BD Biosciences, Franklin Lakes, NJ, USA) and analysed with FlowJo^®^ software (v10, LLC, USA).

### 2.4. Cell Proliferation Assay

Cells were seeded in a 96-well microtiter plate and allowed to adhere overnight. Cells were treated with 100 µg/mL of mAb104-ADC, isotype control IgG1-ADC, mAb104, T-DM-1, trastuzumab and pertuzumab. Cell viability was assessed using the 3-(4,5- dimethylthiazol-2-yl)-5-(3-carboxymethoxyphenyl)-2-(4-sulfophenyl)-2H-tetrazolium (MTS) colorimetric viability assay with MTS as a substrate (Promega^®^, Alexandria, Australia).

### 2.5. In Vivo Studies

To establish xenografts, cells suspended in 50% volume Matrigel^®^ (BD Biosciences) were injected subcutaneously in the right flank of 6-8 week-old female NOD-SCID-IL2R-/- mice (NSG; Austin BioResources Facility, Victoria, Australia). Patient-derived tumour samples (KCC-P-4066) were surgically implanted into the mammary fat pad of mice. Tumour volume (TV) was calculated by the formula [(length × width^2^)/2] where length was the longest axis and width the measurement at right angles to length. Tumours were allowed to establish to approximately 80–100 mm^3^ in size, and mice were then randomised into treatment groups. Treatments were administered at doses indicated via intraperitoneal (i.p.) injections, or intravenously (i.v.) via tail vein as indicated for each study. For all studies, monoclonal antibodies (isotype control IgG1, mAb104 and trastuzumab) were administered three times per week for three weeks, while all ADCs (isotype control IgG1-ADC and mAb104-ADCs) were administered twice per week for three weeks, and T-DM1 was given once or every three weeks, where study length is suitable. Tumour xenograft tissues were resected at designated time points for pharmacodynamic assessments.

All animal studies were approved by the Austin Health Animal Ethics Committee in April 2019 (Protocol #A2019/05605) and conducted in compliance with the Australian Code of Practice for the care and use of animals for scientific purposes.

### 2.6. Dose-Finding Studies for mAb104 Conjugated with Different Payloads

The initial in vivo dose finding for mAb104-MMAE and mAb104-DM1 was conducted as a multi-dose, dose escalation study in female NSG mice bearing established HER2 overexpressing NCI-N87 carcinoma xenografts with five mice per group. ADC-conjugated antibodies (mAb104-MMAE and mAb104-DM1) were administered at increasing doses of 0.5, 1.5 and 3 mg/kg. ADC controls (IgG1-MMAE and IgG1-DM1) were given at 3 mg/kg.

The mAb104-DX8951 dose finding study was conducted as a staggered, single IP injection of monoclonal antibodies (isotype control IgG1, mAb104) or a single IV tail vein injection of mAb104-DX8951 with escalating doses from 0.5, 1, 2, 4 and 10 mg/kg. There were 5 mice in each dose group. Tumour growth was monitored, and the overall mouse health was assessed immediately and, thereafter, antibody administration for adverse effects and tolerability based on approved clinical scores.

### 2.7. Therapy Studies for mAb104 Conjugated with Different Payloads

Using the optimal dose determined from the dose-finding studies, the therapeutic efficacy of mAb104 conjugated to MMAE, DM1 or DX8951 was assessed in the HCC1954 breast cancer xenograft model. Dosing and administration intervals for the naked mAb104 antibody were as per previous in vivo studies evaluating mAb104 as a naked antibody [7]. The dose of T-DM1 was used as previously described in the literature [11,12].

Groups of 5 mice were treated with 20 mg/kg monoclonal antibody (IgG1, mAb104 or trastuzumab), 10 mg/kg T-DM1, 3 mg/kg mAb104-MMAE/DM1 or 4 mg/kg mAb104-DX8951. Control ADCs (IgG1-MMAE/DM1/DX8951) were given equivalent doses as their ADC counterparts. Tumour growth was monitored until reaching endpoints as per our ethics criteria. Anti-tumour activity of mAb104-DX8591 in different tumour models with different HER2 expression.

The therapeutic efficacy of mAb104-DX8951 was further assessed in the xenograft model with high HER2 expression (NCI-H2170) or low HER2 expression (NCI-H1650) and a patient-derived PDX breast cancer model expressing high HER2. Groups of 10 mice were treated with 20 mg/kg monoclonal antibody (IgG1, mAb104 or trastuzumab), 10 mg/kg T-DM1, 4 mg/kg mAb104-DX8951 or IgG1-DX8951. Tumours from 2 mice/group were resected after 2 weeks (6 doses of monoclonal antibodies or 4 doses of ADCs) and at the end of therapy for analysis. For the remaining mice, tumour growth was monitored until reaching endpoints as per our ethics criteria.

### 2.8. Immunohistochemistry

Pharmacodynamic studies by immunohistochemistry (IHC, São Paulo, Brazil) analysis were undertaken on collected tumour xenografts to explore treatment effects on tumour cell proliferation (Ki67), marker of DNA damage phospho-Histone H2A.X (Ser139), (pH2A.X) and marker of apoptosis cleaved caspase 3. Briefly, 5 µm sections of formalin-fixed paraffin-embedded tumour tissues were boiled in 10 mM citrate buffer for antigen retrieval, endogenous peroxidase quenched, and non-specific protein binding blocked in 5% bovine serum albumin (BSA) before probing overnight at 4 °C with anti-pH2A.X (Cell Signalling Technologies #9718) and anti-cleaved caspase 3 (Cell Signalling Technologies #9661) antibodies. Anti-rabbit-HRP (Dako) was added before DAB (Abcam) incubation to detect positive binding. Sections were counterstained with hematoxylin. Tumour necrosis was assessed from H&E-stained sections using HALO tumour classifier, where % tumour necrosis = (necrosis area/total area) × 100. pH2A.X copies were quantified using Indica Labs ISH algorithm (version 4.1.3) per mm^2^ viable tumour area. % cleaved caspase 3 positivity = (positivity/analyzed tumour area) × 100, % ki67 positivity = (ki67 positive cells/total cells) × 100 from 5 images at 20× magnification (>2000 cells). Ki-67 and H&E IHC were performed by the Austin Health pathology department. All slides were scanned using the Aperio Scanner AT2 and images analysed with ImageScope and/or HALO software.

### 2.9. Western Blot Analysis

Tumours collected at week 2 and 3 of treatment and at the end of the study were analysed by Western blot for effects on HER2 and known HER2-activated signalling pathways as previously described (6). In brief, lysed tumour samples were subjected to sodium dodecyl sulphate polyacrylamide gel electrophoresis and immunoblotted with antibodies against HER2 (#2242), HER2 pTyr1221/1222 (#2243), AKT (#4691), AKT pSer473 (#4060), ERK (#4695), ERK pThr202/Tyr204 (#4370) (Cell Signalling Technology^®^, Danvers, MA, USA), and GAPDH (Sigma Aldrich #G5494). Bands were visualised using Clarity Max ECL reagents (Biorad, Hercules, CA, USA) and images captured and analysed with the digital Bio-Rad gel doc system.

### 2.10. Statistical Analysis

Data are presented as the mean ± standard error of the mean (SEM) unless indicated otherwise. All statistical analyses were performed using Prism (versions 8.2 and 10, GraphPad Software). An unpaired *t*-test or one-way ANOVA was used to compare between two groups or multiple comparisons, respectively. *p* < 0.05 was considered significant.

## 3. Results

### 3.1. Generation of mAb104-ADCs

The mAb104-MMAE, mAb104-DM1 and mAb104-DX8951 ADCs and corresponding control IgG1 ADCs were received from Levena Biopharma (San Diego, CA, USA). Calculated Drug Antibody Ratios (DARs) were 3.4 to 3.7 for MMAE and DM-1 conjugates and ~8 for DX8951 conjugates. The DARs are highly comparable to T-DXd and T-DM1, which have been reported as 7.7 and between 3 and 3.6, respectively [11,13].

### 3.2. Mab104-ADC Binding

**Figure 1:**
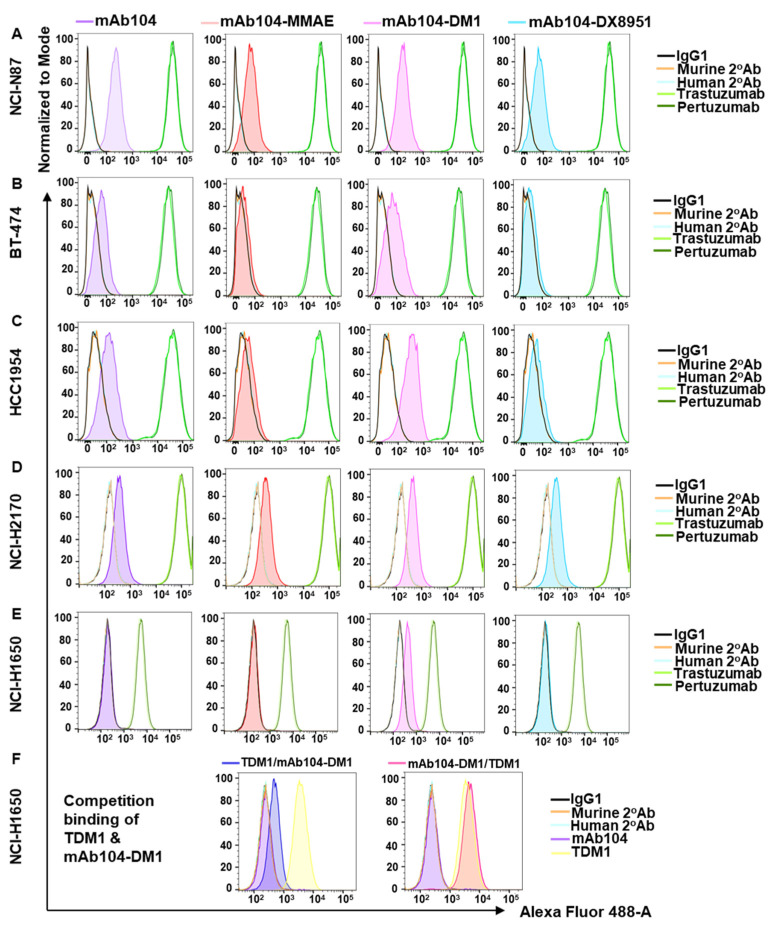
**FACS analysis highlights differing levels of HER2 in breast and gastric cell lines.** FACS analysis showing a small shift in mAb104 in high HER2-expressing NCI-N87 (**A**), BT-474 (**B**), HCC1954 (**C**) and NCI-H2170 (**D**) but not in low HER2-expressing NCI-H1650 (**E**) cell lines (in the first column). Conjugation of cytotoxic payloads MMAE (second column), DM1 (third column), and DX8951 (fourth column) has no impact on the binding of mAb104-ADCs in high HER2-expressing cancer cell lines. (**F**) Competition FACS assay showing mAb104-DM1 binding (E, third column) is not affected by pre-incubation of TDM1 and vice versa. Data representative of >2 independent experiments.

To verify binding properties to HER2 following conjugation of antibody to the cytotoxic payloads, flow cytometry analyses were performed in a panel of cell lines expressing a range of HER2 levels (Figure 1). The FACS analyses confirmed equivalent HER2 reactivity of the parental mAb104 antibody and ADC formats of mAb104. Pertuzumab and trastuzumab demonstrated a larger log shift in all cell lines compared to mAb104 and mAb104-ADCs, which is consistent with our previous work, indicating that at least in vitro, the mAb104-epitope is exposed in a subset of HER2 receptors [7]. Using flow cytometry on NCI-H1650 cells, we investigated whether mAb104-DM1 and T-DM1 compete for endogenous HER2. Pre-incubating cells with either mAb104-DM1 or T-DM1 did not affect the binding of the other drug to the cell surface HER2. These results are consistent with our previous findings that the mAb104 epitope is distinct from that of trastuzumab [7].

### 3.3. Effect of mAb104-ADCs on Proliferation in Vitro

**Figure 2:**
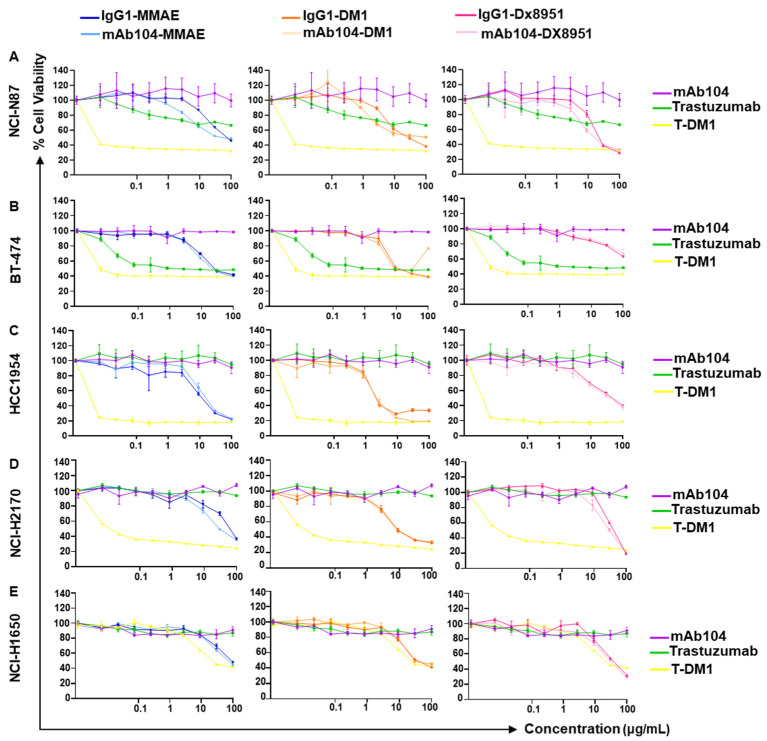
**Proliferative effects of mAB104-ADCs.** The effects of mAb104-ADCs on cell proliferation were assessed in MTS assays in NCI-N87 (**A**), BT-474 (**B**), HCC1954 (**C**), NCI-H2170 (**D**) and NCI-H1650 (**E**). Left column: mAb104-MMAE. Middle column: mAb104-DM1. Right column: mAb104-DX8951. Trastuzumab, pertuzumab and T-DM1 are included as controls. Data show dose-dependent effects of mAb104-ADCs. Data are presented as mean ± SD and are representative of >2 independent experiments.

The anti-proliferation properties of the ADCs were compared to mAb104 and controls in a panel of cell lines expressing a range of HER2 levels (Figure 2). As expected, mAb104-ADCs did not have a significant reduction in cell viability compared to control IgG1 ADCs, given that binding to cell lines is low in vitro, and as shown in our prior data, mAb104 has no activity in vitro but has activity in vivo [7]. The cytotoxicity of mAb104-ADCs in vitro was dose-dependent and increased compared to mAb104, which has no detectable effect in vitro. While lacking in vitro activity, mAb104-ADCs showed potent anti-tumour effects in a variety of xenograft models with differential HER2 expression. We hypothesise this discrepancy is due to differences in HER2 receptor activation, turnover, and internalisation kinetics in vivo compared to in vitro. It can therefore be postulated that in vitro studies may not accurately examine the mechanism of action of this class of antibodies.

### 3.4. Effect of mAb104-ADCs In Vivo

#### 3.4.1. Dose Finding Study

**Figure 3:**
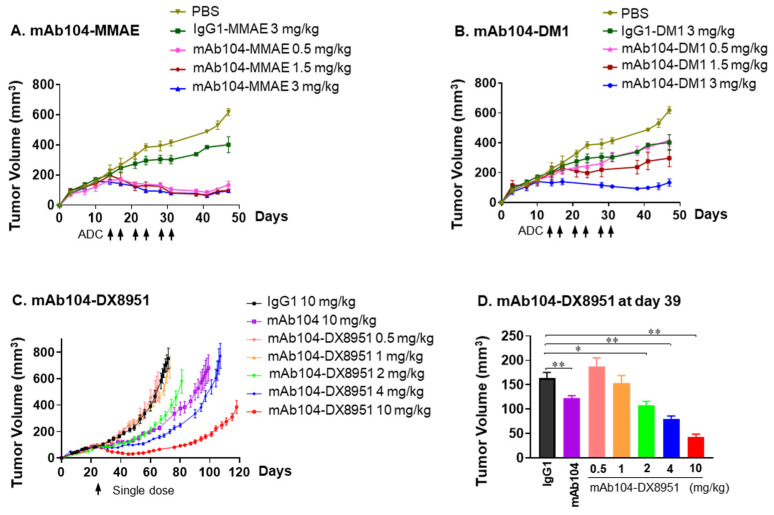
**Dose finding and therapy study of mAb104-ADCs demonstrates significant anti-tumour activity.** In high HER2-expressing gastric xenograft NCI-N87, (**A**) mAb104-MMAE, (**B**) mAb104-DM1, and (**C**) mAb104-DX8951 were administered at the indicated doses and schedule. Control IgG-MMAE and -DM1 were given at 120 mg/kg, and control IgG1-DX8951 and mAb104 at 10 mg/kg. (**D**) significant dose-dependent tumour growth inhibition by mAb104-DX8951 after 2 weeks of therapy. Monoclonal antibodies were given 3 times per week for 3 weeks, or mAb104-ADCs and their respective isotype control twice per week for 3 weeks at the indicated doses. T-DM1 was given as a single dose at the start of therapy. Doses in mg/kg are indicated for mice of 25 g. Data are represented as mean ± SEM. N = 10 mice per group. * *p* < 0.05, ** *p* < 0.01.

The mAb104-MMAE ADC demonstrated equivalent efficacy at all doses assessed in a high HER2-expressing gastric cancer xenograft model, NCI-N87, with significant sustained tumour growth inhibition compared to control IgG1-MMAE (Figure 3A). A dose-dependent growth inhibition was observed for mAb104-DM1 compared to controls (Figure 3B). The anti-tumour efficacy of mAb104-DM1 0.5 mg/kg was equivalent to mAb104-MMAE 3 mg/kg when each was administered twice weekly for 3 weeks.

To establish the safety of the mAb104-DX8951, a staggered dose escalation study of 0.5, 1, 2, 4, or 10 mg/kg of the novel mAb104-DX8951 via a single i.v. injection was conducted in mice bearing established HER2 overexpressing (IHC 3+) NCI-N87 carcinoma xenografts. Dose-dependent inhibition of tumour growth was observed within two weeks of a single dose of mAb104-DX8951, with the 10 mg/kg dose significantly more effective than the 2 and 4 mg/kg ADC doses (*p* = 0.008 and *p* = 0.016, respectively) (Figure 3C,D). The treatments were well tolerated, with the maximum tolerated dose not reached, and no changes in body weight or overall health of the mice were observed in response to therapy (Supplementary Figure S1).

The dose level of 4 mg/kg of mAb104-DX8951 and 3 mg/kg of mAb104-MMAE and mAb104-DM1 was used for further therapy studies.

#### 3.4.2. Efficacy of mAb104-ADCs in a HER2-Positive (IHC 3+) Trastuzumab Resistant Breast Cancer Xenograft Model

**Figure 4:**
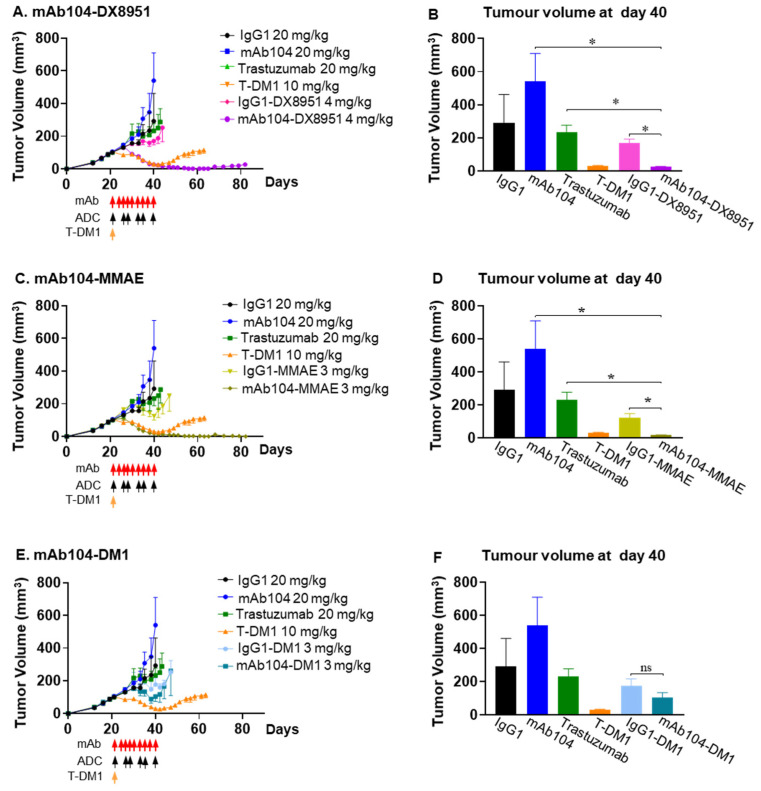
**mAb104-ADCs show tumour growth inhibition.** In trastuzumab-resistant HER2+/ER-/PR- breast cancer xenograft HCC1954, significant complete and durable tumour growth inhibition was observed with mAb104-DX8951 (**A**,**B**) and mAb104-MMAE (**C**,**D**), and mAb104-DM1 to a lesser extent (**E**,**F**). Monoclonal antibodies were given 3 times per week for 3 weeks, or mAb104-ADCs and their respective isotype control twice per week for 3 weeks at the indicated doses. T-DM1 was given as a single dose at the start of therapy. Doses in mg/kg are indicated for mice of 25 g. Data are represented as mean ± SEM. n = 10 mice per group. * *p* < 0.05.

MAb104-DX8951 showed significant tumour growth inhibition compared to ADC control (IgG1-DX8951) (*p* = 0.0022), trastuzumab (*p* = 0.0043) or mAb104 alone (*p* = 0.0043) in the HER2-positive (IHC 3+) trastuzumab-resistant breast cancer xenograft model, HCC1954. Treatment with mAb104-DX8951 resulted in complete and durable responses with the therapeutic benefit lasting longer than the standard of care treatment, T-DM1 (Figure 4A,B). Similar durable anti-tumour benefit was observed in mice treated with mAb104-MMAE ADC compared to all treatments, including T-DM1 (Figure 4C,D). The mAb104-DM1 ADC had a superior anti-tumour effect than the IgG1 control and trastuzumab (Figure 4E,F).

#### 3.4.3. Efficacy of mAb104-DX8951 ADC Therapy in HER-Positive Squamous Cell Lung Carcinoma

**Figure 5:**
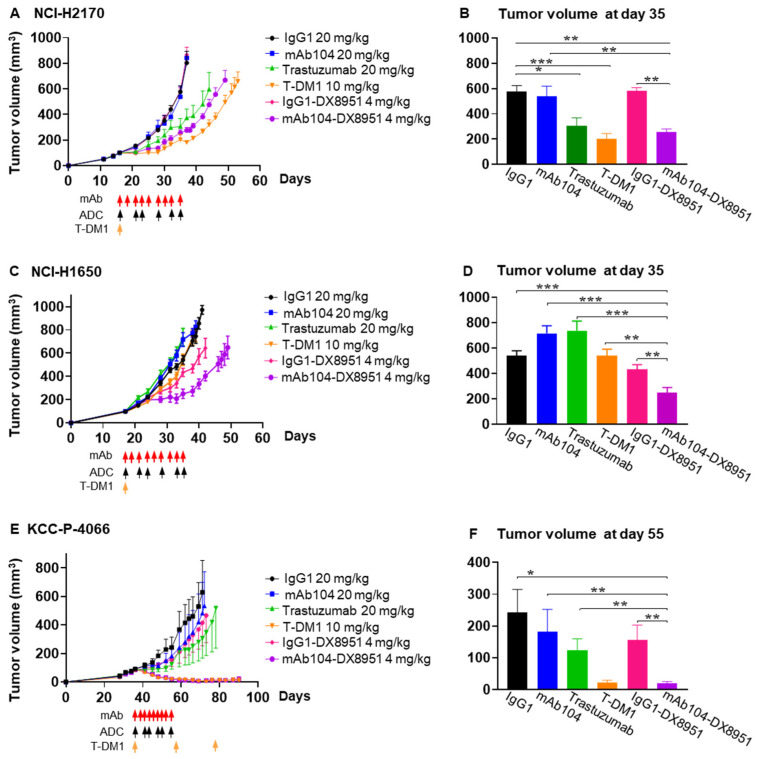
**mAb104-DX8951 is efficacious in NCI-H2170 tumours**. mAb104-DX8951 demonstrate significant anti-tumour activity comparable to T-DM1 in high HER2-expressing lung NCI-H2170 xenograft (**A**,**B)**, in low HER2-expressing lung NCI-H1650 xenograft (**C**,**D**) and KCC-P-4066 breast PDX (**E**,**F**). Mice weighing 20–25 g during the study period were given IP 0.5 mg monoclonal antibodies thrice per week for 3 weeks, and doses in mg/kg are indicated for 25 g mice. T-DM1 was given once every 3 weeks, where applicable (orange arrow), and mAb104-DX8951 and its respective control were given twice weekly for 3 weeks at the indicated doses. Data are represented as mean ± SEM. n = 10 mice per group. * *p* < 0.05, ** *p* < 0.01, *** *p* < 0.001.

Based on the safety and efficacy demonstrated by mAb104-DX8951 ADC, we selected this ADC as our lead compound for further assessment. In the HER2 overexpressing (IHC 3+) NCI-H2170 squamous cell lung carcinoma xenografts [14,15], mAb104-DX8951 resulted in significant tumour growth inhibition compared to the ADC control (IgG1-DX8951) (*p* = 0.0025) and numerically superior to trastuzumab (*p* = 0.533). MAb104-DX8951 ADC demonstrated equivalent efficacy to T-DM1 with no significant differences between the two treatment arms (Figure 5A,B).

#### 3.4.4. Efficacy of mAb104-DX8951 ADC Therapy in EGFR Mutant, HER2 Low Adenocarcinoma Xenograft

The NCI-H1650 lung carcinoma cell line harbours the EGFR exon 19 deletion (DelE746-A750) and is resistant to EGFR tyrosine kinase inhibitors [16]. NCI-H1650 cells express high levels of EGFR (>150,000 EGFR receptors per cell) but approximately 10-fold lower HER2 expression (~50,000 HER2 receptors per cell) [17]. Compared to all treatment arms, including trastuzumab, ADC control (IgG1-DX8951) and T-DM1, mAb104-DX8951 therapy resulted in significant anti-tumour effects (*p* < 0.007) (Figure 5C,D). The median survival of the mAb104-DX8951 cohort was 55 days, significantly longer than that of other groups, including the T-DM-1 median survival of 41.5 days (*p* = 0.0006) (Table 1).

#### 3.4.5. Efficacy of mAb104-DX8951 ADC Therapy in HER2-Positive (IHC 2/3+), ER-Positive Breast Cancer PDX

Mab104-DX8951 resulted in significant and durable anti-tumour effect compared to control IgG1 ADC (*p* = 0.007) and naked antibody controls (*p* = 0.0002) and has equivalent efficacy to T-DM1 (Figure 5E,F).

## 4. Pharmacodynamic Studies

Tumors taken at week 2 and 3 of treatment and at end of study were analyzed by IHC for percentage necrosis present, pH2AX DNA damage, apoptosis marker (cleaved caspase 3), and Ki67 proliferation marker. In both NCI-H2170 and NCI- H1650 NSCLC xenografts mAb104-DX8951 did not induce apoptosis or increase necrosis compared to the control arms (Supplementary Figures S2 and S3). In addition, tumors from NCI-H2170 were taken at week 2 and 3 of treatment and analyzed by Western blot for effects on HER2 and known HER2-activated signaling pathways. Treatment with mAb104 resulted in reduction of total HER2 and phosphorylated HER2 expression, mAb104-DX8951 reduced phosphorylated HER2 expression but not total HER2 (Supplementary Figure S4 for NCI-H2170 and Figure S5 for NCI-H1650).

Due to the efficacy of treatment achieved with the 104 ADCs in the PDX and HCC1954 models there was insufficient tumor tissue from the 104-DX8951 arms to conduct these analyses.

## 5. Discussion

Antibody drug conjugates of mAb104 have been stably produced with retention of HER2 binding and in vitro cytotoxic specificity following conjugation via linkers to MMAE, DM1 and DX8951. In vivo studies demonstrate that mAb104 ADCs have significant efficacy in various tumour models with variable HER2 expression. Trastuzumab has been the cornerstone of HER2-directed therapy in HER2-overexpressing breast and gastric cancer; however, resistance to trastuzumab or trastuzumab conjugate regimens is universal [18]. In our study, mAb104-DX8951 showed significant tumour growth inhibition in a trastuzumab-resistant breast cancer model, compared to ADC control, with the therapeutic benefit lasting longer than the standard of care treatment, trastuzumab–emtansine (T-DM1). The ability of mAb104 ADCs to target a conformational epitope exposed in tumour-specific conditions allows effective and specific tumour targeting and potentially reduced toxicity. In our studies, mAb104-ADC treatments were well tolerated, and no changes in body weight were observed in response to therapy.

In NSCLC, various novel ADCs targeting oncogenic targets, including HER2, HER3, Trophoblast cell-surface antigen 2 (TROP2), MET, NECTIN4, Tissue Factor (TF), Carcinoembryonic antigen-related cell adhesion molecule 5 (CEACAM5), mesothelin, and LIV1, are in clinical trials [3]. The HER2-directed ADCs T-DM1 and trastuzumab deruxtecan (T-DXd) have been investigated in patients with EGFR-mutated NSCLC and HER2 overexpression [19,20]. The combination of T-DM1 and osimertinib in patients with EGFR-mutated NSCLC who had progressed on prior EGFR-tyrosine kinase inhibitor (TKI) therapy, including osimertinib, demonstrated a favourable safety profile, albeit with limited clinical efficacy [19]. In contrast, T-DXd has received regulatory approval in several jurisdictions for the treatment of HER2-mutant NSCLC in patients who have received a prior systemic therapy. Ongoing clinical trials are evaluating T-DXd as monotherapy as well as in combination with immunotherapy and chemotherapy in patients with HER2-overexpressing NSCLC. These studies include patient populations with EGFR-mutated NSCLC who have been previously treated with EGFR-TKIs (DESTINY-Lung01 and NCT04042701) [21,22]. In our study, mAb104-DX8951 demonstrated a durable anti-tumour response in an Osimertinib-resistant EGFRmt NSCLC model, a tumour type with an unmet clinical need. Intriguingly, this response was seen despite the model having low-level expression of HER2. This suggests a potential role for mAb104-ADCs in settings beyond classical HER2 overexpression, a growing area of interest in the context of HER2-low cancers.

In general, expression levels and distributional patterns of tumour antigens correlate with ADC response [23], as seen with T-DM1, where higher levels of HER2 expression were associated with better responses [24]. However, the efficacy of certain ADCs, such as T-DXd, is not solely dependent on high levels of target antigen expression. This has been attributed to the specific characteristics of the target antigen, including binding affinity and rate of internalisation [25]. This is observed with T-DXd in NSCLC, where its therapeutic activity is primarily directed against HER2 mutations, rather than amplification or overexpression, which are the more traditional HER2 targets [20]. Moreover, certain payloads employed in these ADCs can elicit an anti-tumour response in cells that do not express the target antigen, through the bystander effect. The clinical significance of this effect was demonstrated in the DESTINY-Breast04 trial, where T-DXd significantly improved progression-free and overall survival in patients with HER2-low metastatic breast cancer. [26]. However, unlike in the DESTINY-Breast04 trial, much lower responses were seen in patients with HER2-low (IHC 2+) gastric or metastatic colorectal cancer when treated with T-DXd [27,28]. These findings highlight some of the challenges of HER2 expression as a predictive biomarker due to the spatial and temporal intratumour heterogeneity of HER2, the lack of standardised and site-specific scoring systems beyond breast and gastric cancer, as well as the impact of concomitant oncogenic drivers on ADC activity [29]. Despite the mAb104-epitope not being detected in low HER2 expressing cell lines by FACS, we postulate that mAb104 selectively interacts with distinct sub-populations and conformations of HER2 depending on the intra-tumoural conditions in the tumour, as we have demonstrated with an antibody to a similar conformationally exposed epitope of EGFR in animal models and in patients [30]. This selective interaction enables the delivery of the payload and allows the payload to exert antitumour activity through direct and bystander effects. This would explain the significant anti-tumour activity of mAb104-DX8951 observed in models with low HER2 expression.

Our study utilised T-DM1 as a comparator, which is an approved HER2 ADC with established efficacy in breast cancer. However, we acknowledge the absence of direct comparative studies with T-DXd, which utilises a topoisomerase I inhibitor payload (similar class to DX8951) and a cleavable linker, which has been approved for previously treated HER2-positive solid tumours.

## 6. Conclusions

In conclusion, we have designed a novel HER2-targeting ADC, mAb104-DX8951, which has shown great potential to respond to trastuzumab-resistant HER2-positive cancers, low HER2-expressing cancers, as well as HER2 overexpressing cancers. Moving forward, rational combination strategies with immune checkpoint inhibitors and anti-angiogenic therapies will likely be important to augment the activity of mAb104-DX8951 and overcome potential mechanisms of resistance.

## Figures and Tables

**Table 1:** Survival analysis of NCI-H1650 tumour-bearing mice treated with monoclonal antibodies (control isotype antibody IgG1, mAb104 and trastuzumab) or ADCs (T-DM1, IgG1-DX8951 or mAb104-DX8951). Median survival is shown in days. Statistics analysis performed by log-rank (Mantel–Cox) test using Prism v10. P values for comparisons between mAb104-DX8951 and other treatments.

Table	Median Survival (Days)	*p* Value
IgG1	41	0.0008
Mab104	40	0.0008
Trastuzumab	39.5	0.0007
T-DM1	41.5	0.0006
IgG1-DX8951	48	0.0223
Mab104-DX8951	55	

## Data Availability

The data generated in this study are available upon request from the corresponding author.

## Supplementary Materials

The following supporting information can be downloaded at: https://www.mdpi.com/article/10.3390/cancers17182995/s1. Figure S1: Body weight of mice treated with mAb104-ADCs; Figure S2: Pharmacodynamic analysis by Immunohistochemistry of necrosis, pH2A.X, cleaved caspase 3 and ki67 in lung cancer xenograft NCI-H2170; Figure S3: Pharmacodynamic analysis by Immunohistochemistry of necrosis, pH2A.X, cleaved caspase 3 and ki67 in lung cancer xenograft NCI-H1650; Figure S4: Pharmacodynamic analysis by Western blot of mAb104-DX8951 effects on HER2 and known HER2 effector pathways in NCI-H2170 xenograft tumours; Figure S5: Pharmacodynamic analysis by Western blot of mAb104-DX8951 effects on HER2 and known HER2 effector pathways in NCI-H1650 xenograft tumours.
