# Supplementary material for: Characterisation of mAb104 Antibody–Drug Conjugates Targeting a Tumour-Selective HER2 Epitope
Source: Cancers (Basel). 2025 Sep 13;17(18):2995. doi: 10.3390/cancers17182995 (PMC12468726; doi:10.3390/cancers17182995)
Supplement: Supplementary file 1 [file cancers-17-02995-s001.zip › cancers-3858750-supplementary.pdf]

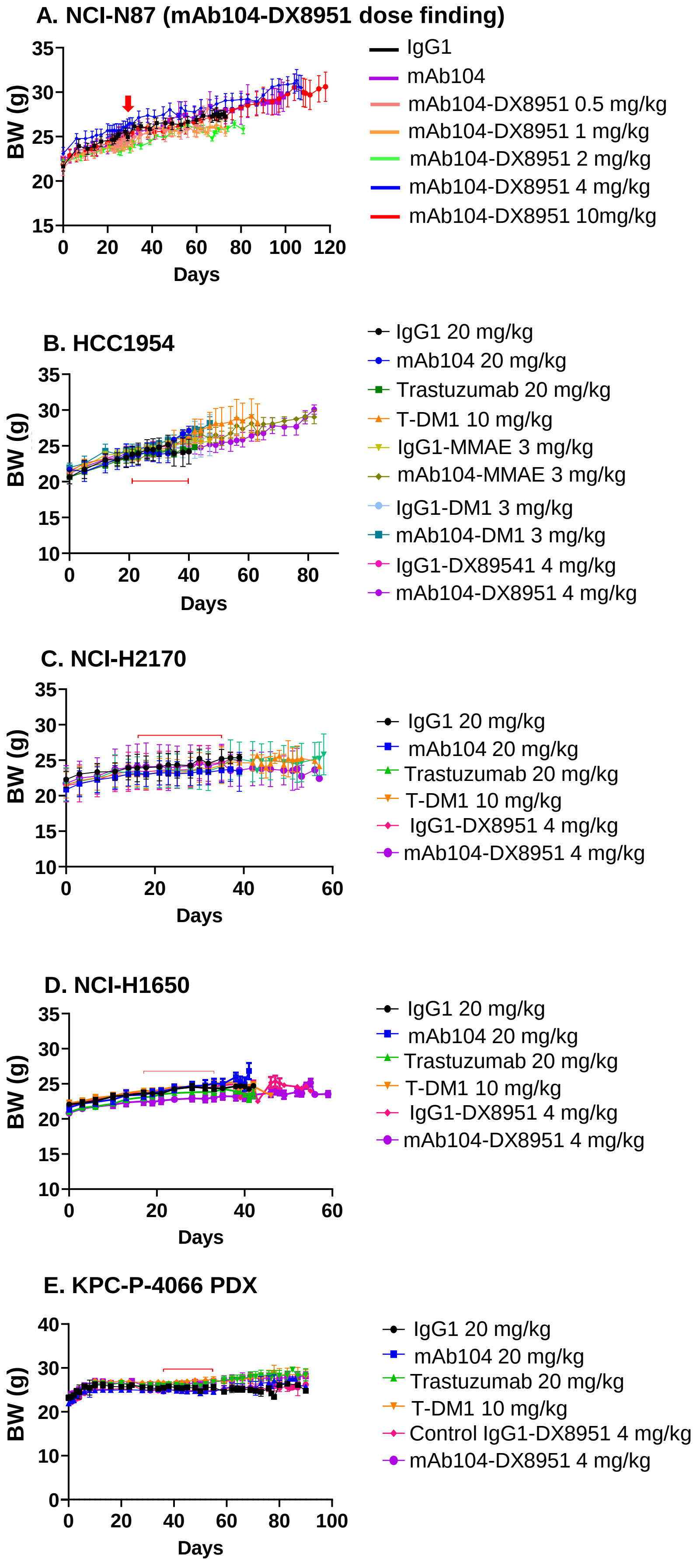

Figure S1: Body weight of mice treated with mAb104-ADCs

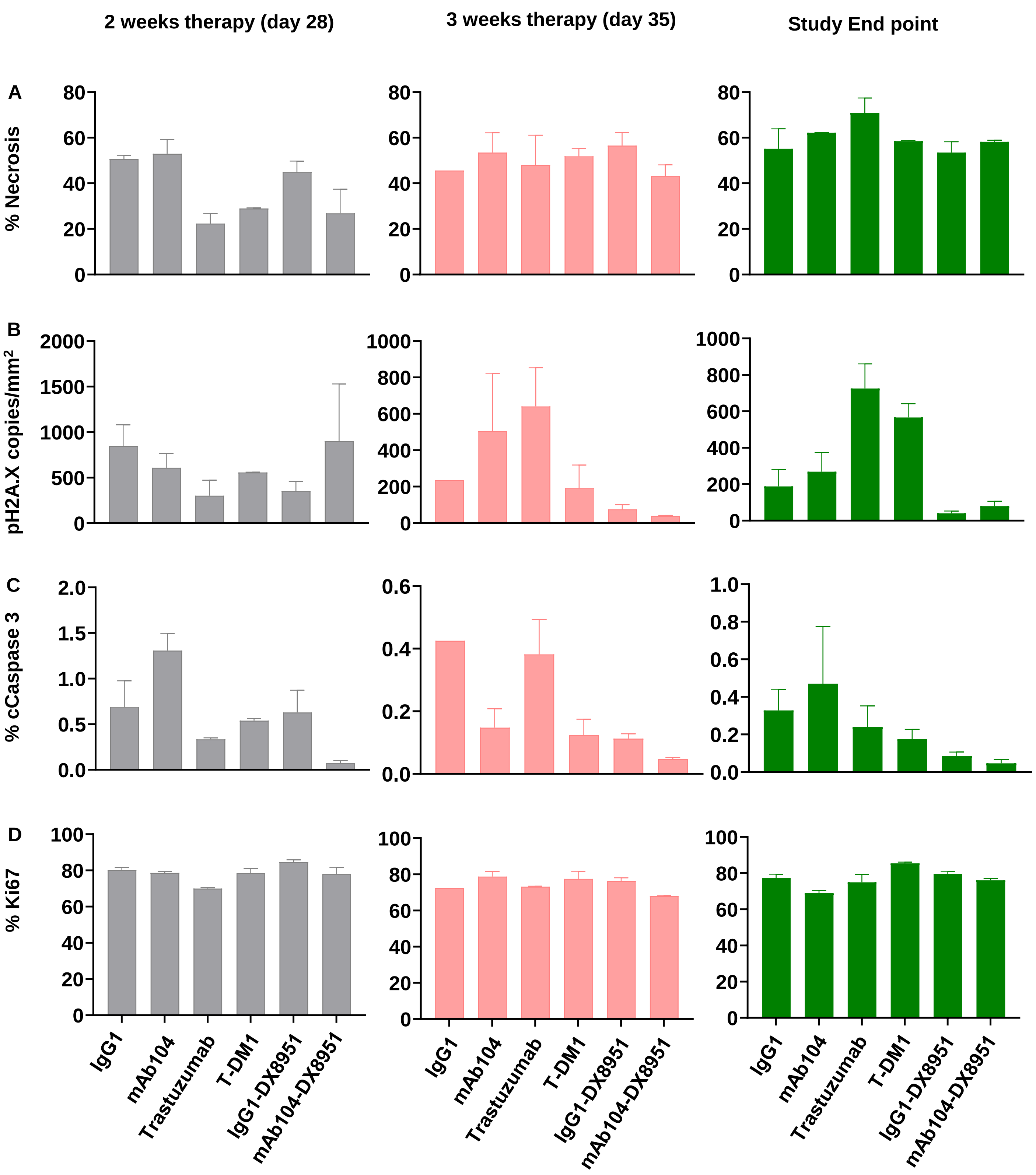

Figure S2: Pharmacodynamic analysis by Immunohistochemistry of necrosis, pH2A.X, cleaved caspase 3 and ki67 in lung cancer xenograft NCI-H2170

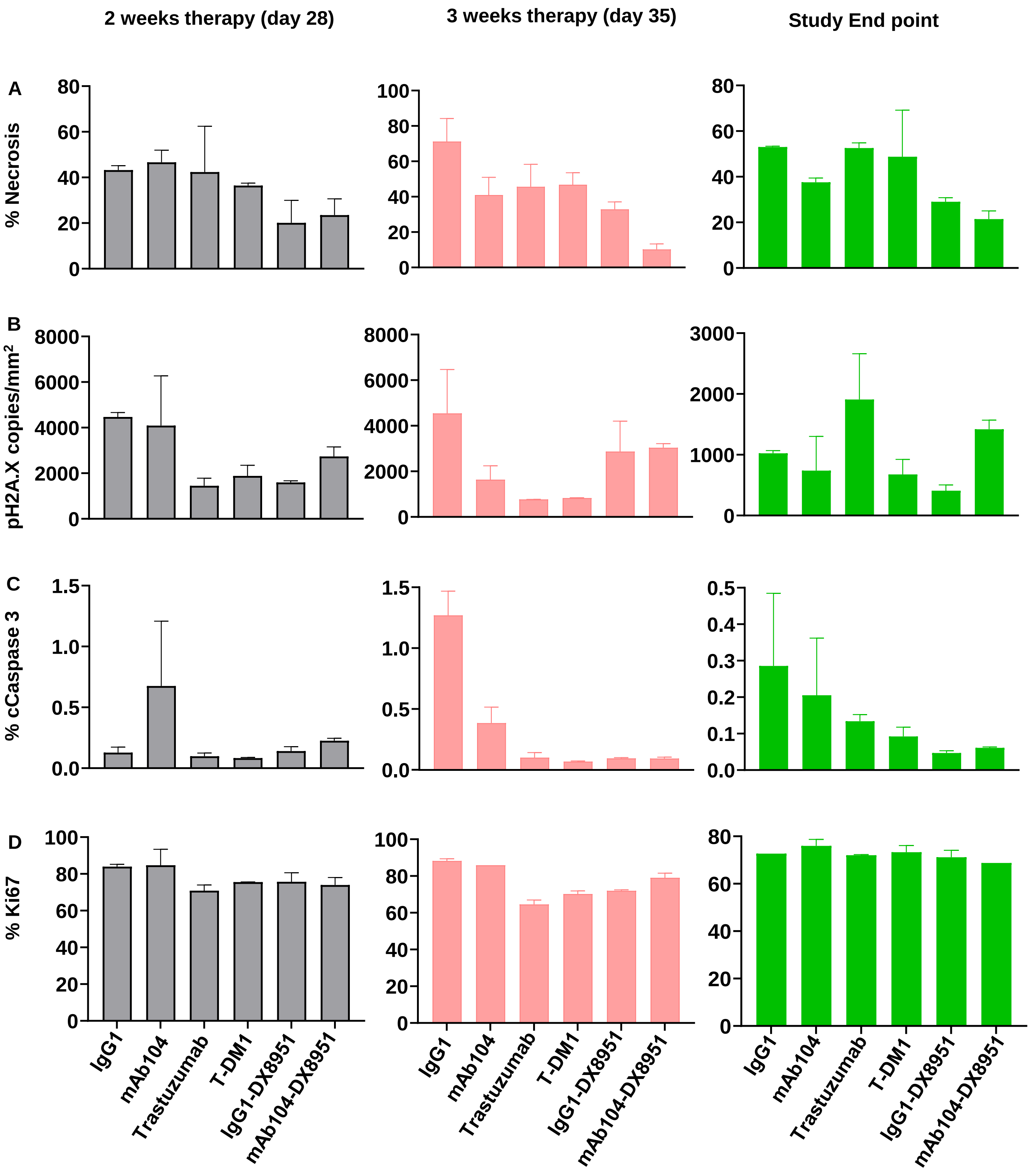

Figure S3: Pharmacodynamic analysis by Immunohistochemistry of necrosis, pH2A.X, cleaved caspase 3 and ki67 in lung cancer xenograft NCI-H1650

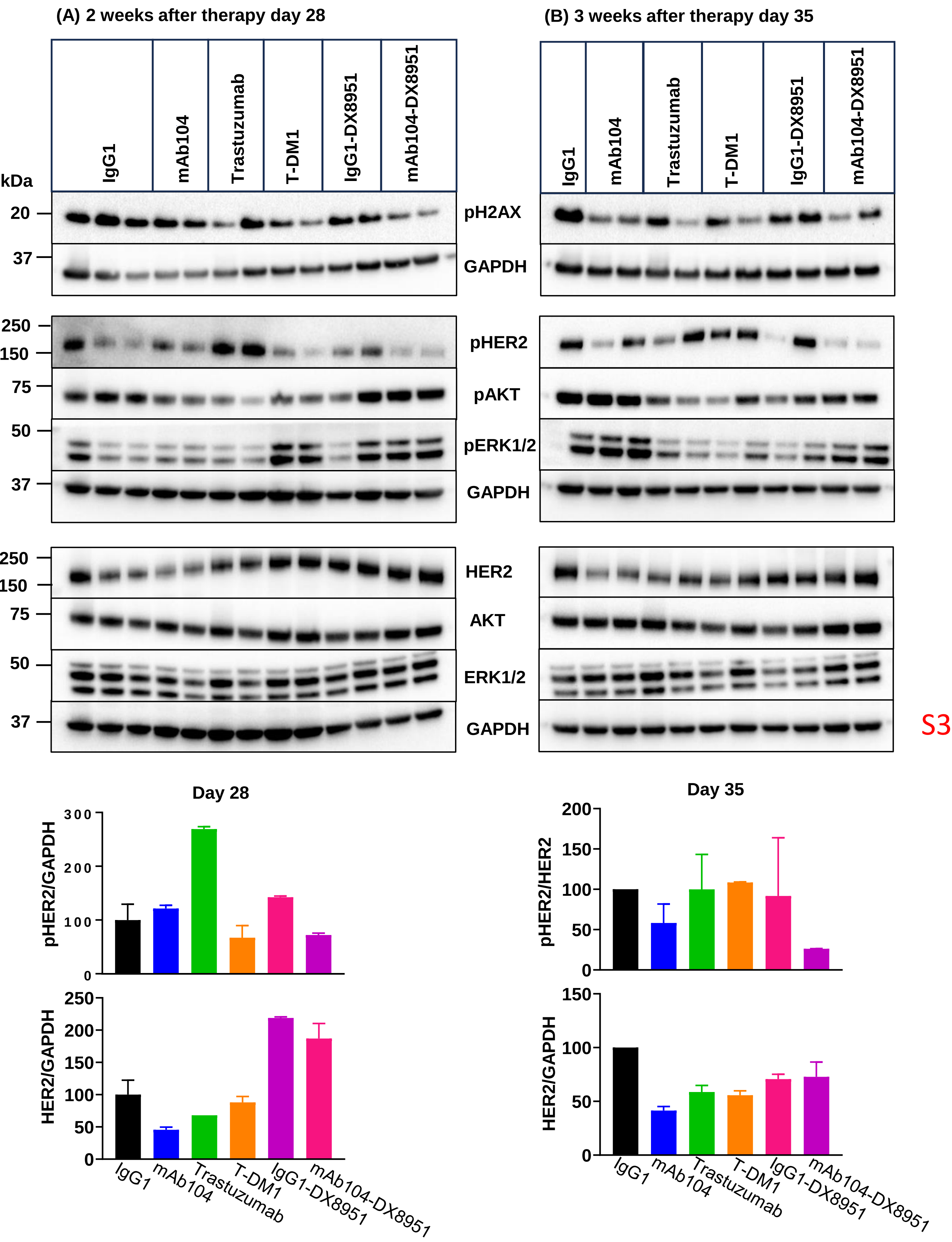

S3

Figure S4: Pharmacodynamic analysis by Western blot of mAb104-DX8951 effects on HER2 and known HER2 effector pathways in NCI-H2170 xenograft tumours

Supplementary Figure. Uncropped blots and densitometry analysis for **Figure S4 (A)** for pH2AX, pHER2, pAKT, pERK1/2, GAPDH, HER2, AKT, ERK1/2 from NCI-H2170 week 2 tumor samples.

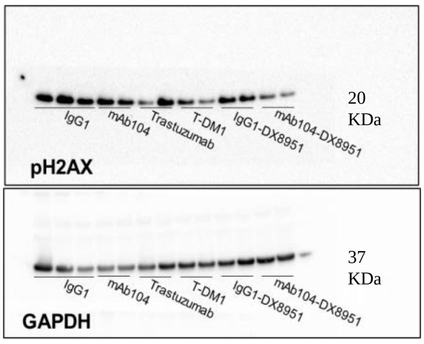

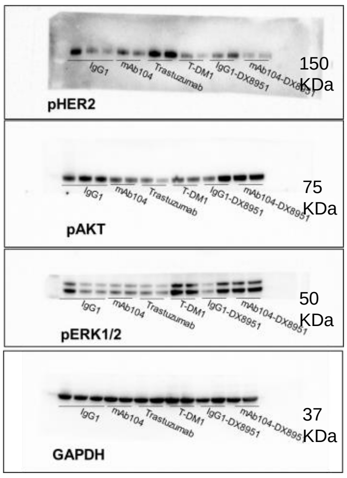

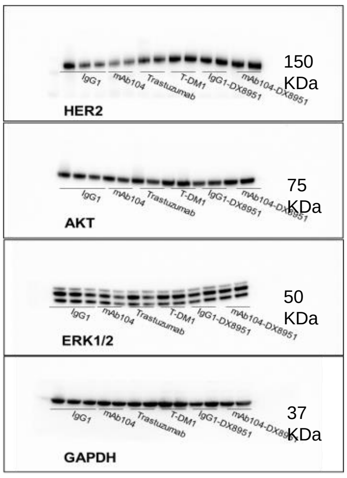

	pH2AX/GAPDH
IgG1	0.3627
	0.8205
	0.9478
mAb104	0.8250
	0.5122
Trastuzumab	0.1949
	0.5210
TDM1	0.2504
	0.1697
IgG1-DX8951	0.4208
	0.2838
mAb104-DX8951	0.1564
	0.0909

	pHER2/GAPDH	pAKT/GAPDH	pERK/GAPDH
IgG1	0.0959	0.3514	0.4267
	0.0510	0.5562	0.2290
	0.0364	0.4445	0.1769
mAb104	0.0778	0.2472	0.1885
	0.0705	0.2393	0.2396
Trastuzumab	0.1617	0.2050	0.1845
	0.1671	0.0886	0.1362
TDM1	0.0548	0.2382	0.5389
	0.0274	0.2451	0.4580
IgG1-DX8951	0.0857	0.3143	0.1943
	0.0884	0.5616	0.4492
mAb104-DX8951	0.0420	0.6430	0.5400
	0.0462	0.7676	0.7381

	HER2/GAPDH	AKT/GAPDH	ERK/GAPDH
IgG1	2.3174	1.2292	2.8897
	1.4254	1.3123	2.5305
	1.1079	0.8562	1.5294
mAb104	0.6725	0.8235	1.3338
	0.8022	0.7221	0.8493
Trastuzumab	1.0993	0.8517	1.3590
	1.0989	0.6886	0.9525
TDM1	1.2755	0.7740	1.1666
	1.5714	0.9648	1.4327
IgG1-DX8951	2.6453	1.1770	2.0286
	3.3991	1.5720	3.1430
mAb104-DX8951	3.5652	2.1515	3.0390
	3.5154	1.9620	2.8936

Supplementray Figure. Uncropped blots and densitometry analysis for **Figure S4 (B)** for pH2AX, pHER2, pAKT, pERK1/2, GAPDH, HER2, AKT, ERK1/2 from NCI-H2170 week 3 tumor samples.

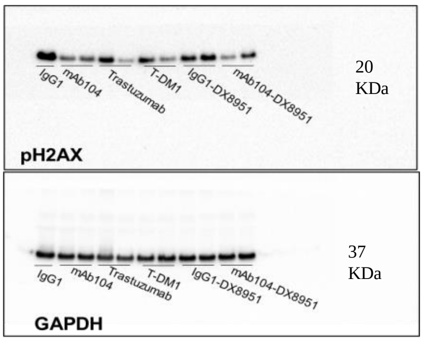

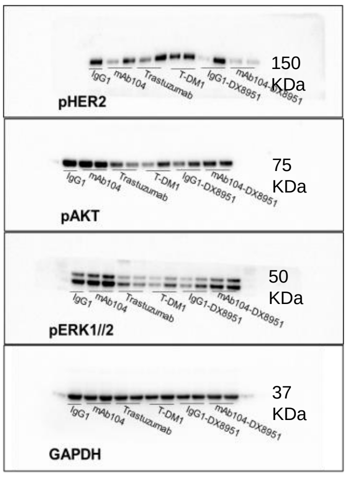

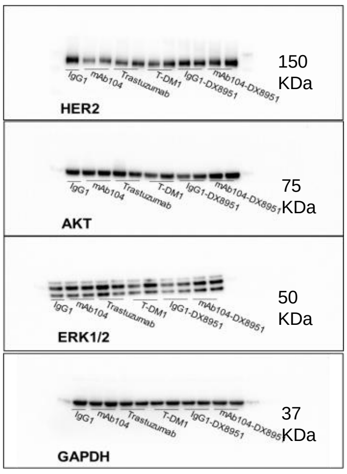

	pH2AX/GAPDH
IgG1	0.5718
mAb104	0.1608
	0.1860
Trastuzumab	0.3213
	0.1092
TDM1	0.3037
	0.1214
IgG1-DX8951	0.2622
	0.3472
mAb104-DX8951	0.1052
	0.2018

	pHER2/GAPDH	pAKT/GAPDH	pERK/GAPDH
IgG1	0.2813	0.5841	0.2961
mAb104	0.0973	0.7613	0.4412
	0.2300	0.5268	0.4652
Trastuzumab	0.1590	0.2909	0.1927
	0.4028	0.2048	0.1381
TDM1	0.3033	0.1768	0.0975
	0.3068	0.3179	0.1754
IgG1-DX8951	0.0550	0.2333	0.1200
	0.4610	0.3503	0.2157
mAb104-DX8951	0.0743	0.3835	0.3092
	0.0738	0.3955	0.4030

	HER2/GAPDH	AKT/GAPDH	ERK/GAPDH
IgG1	1.4970	0.4335	0.6445
mAb104	0.5650	0.3817	0.7176
	0.6761	0.3614	0.6673
Trastuzumab	0.7871	0.4206	1.0721
	0.9710	0.3226	0.7977
TDM1	0.7718	0.2550	0.6026
	0.8954	0.3100	0.8340
IgG1-DX8951	1.1255	0.2857	0.4909
	0.9926	0.3204	0.5890
mAb104-DX8951	0.8840	0.3718	0.7283
	1.2961	0.4979	0.8006

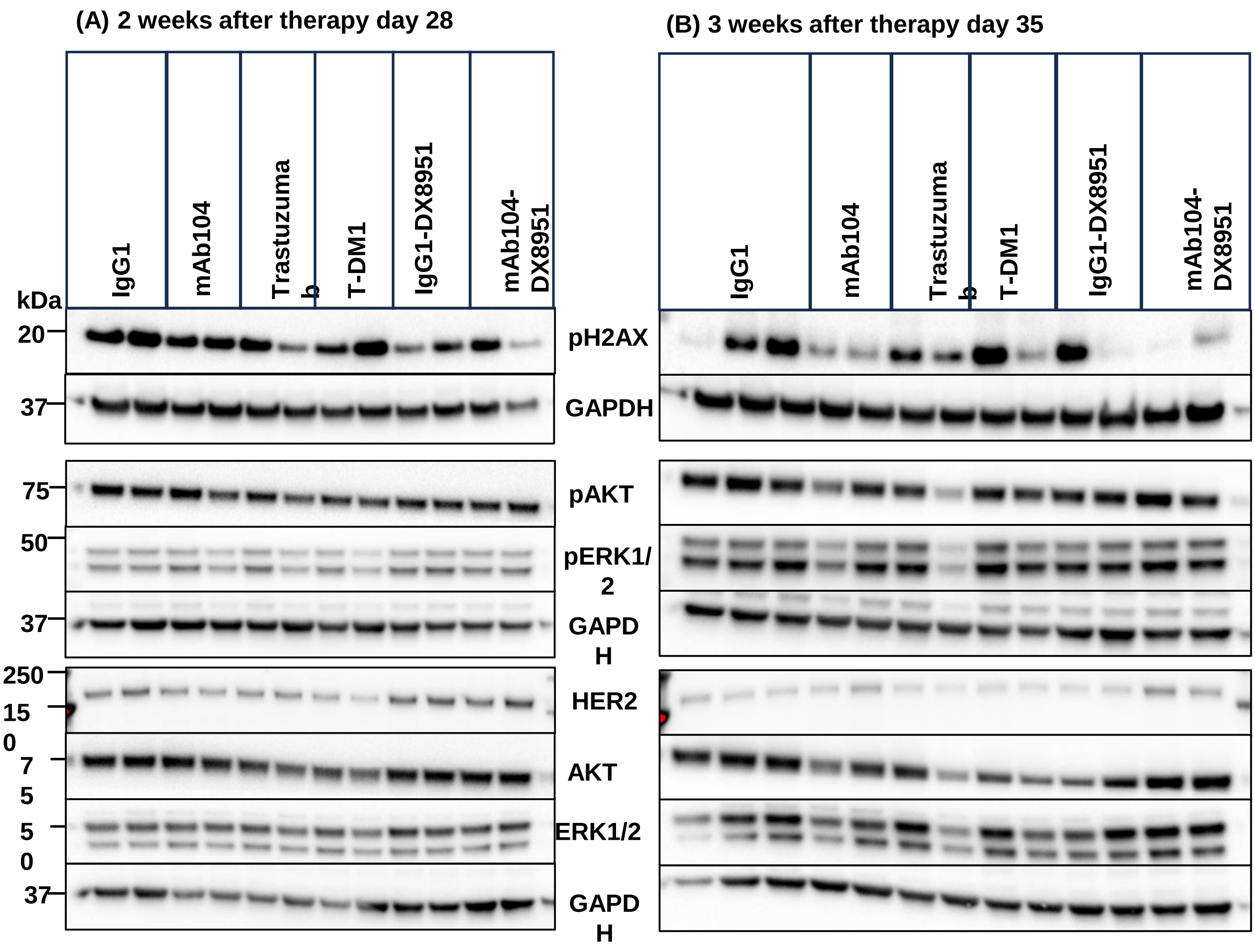

Figure S5: Pharmacodynamic analysis by Western blot of mAb104-DX8951 effects on HER2 and known HER2 effector pathways in NCI-H1650 xenograft tumours

Supplementary Figure. Uncropped blots and densitometry analysis for **Figure S5 (A)** for pH2AX, pAKT, pERK1/2, GAPDH, HER2, AKT, ERK1/2 from NCI-H1650 week 2 tumor samples.

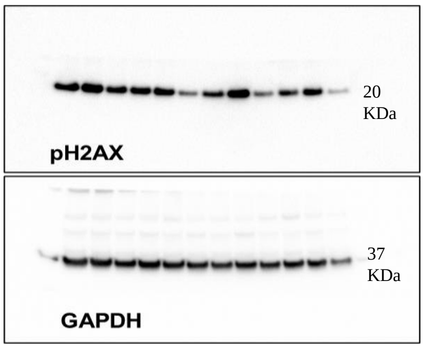

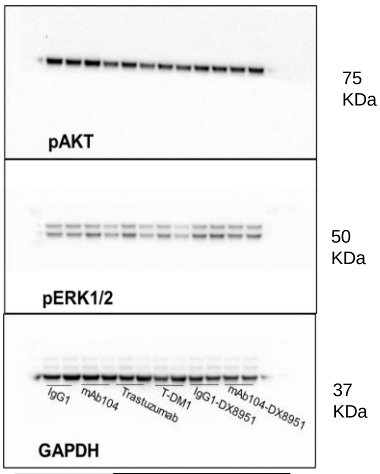

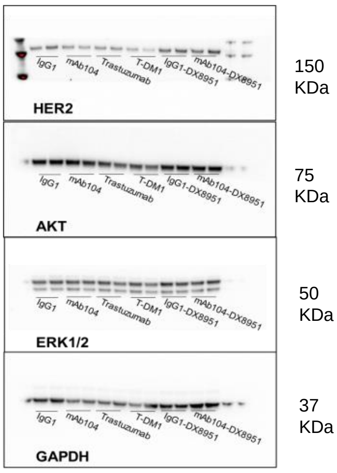

	pH2AX/GAPDH
IgG1	0.8850
	1.3204
104	0.7836
	0.7565
Tras	0.8319
	0.3283
TDM1	0.7328
	1.3777
IgG1-DX8951	0.3966
	0.5420
mAb104-DX8951	0.8605
	0.2426

	pAKT/GAPDH	pERK/GAPDH
IgG1	0.2029	0.4731
	0.1389	0.3508
104	0.1821	0.4232
	0.1320	0.3431
Tras	0.1716	0.5388
	0.1208	0.3163
TDM1	0.1822	0.5172
	0.1336	0.2290
IgG1-DX8951	0.1869	0.5618
	0.1995	0.7252
mAb104-DX8951	0.2027	0.6123
	0.2530	0.7092

	HER2/GAPDH	AKT/GAPDH	ERK/GAPDH
IgG1	0.4792	0.5258	1.3775
	0.5522	0.5338	1.3236
104	0.6200	0.8733	2.1737
	0.4190	0.6764	1.7947
Tras	0.5848	0.6848	2.1863
	0.5405	0.5306	1.6215
TDM1	0.5928	0.8485	3.0790
	0.2105	0.3743	1.3171
IgG1-DX8951	0.5961	0.5267	1.6991
	0.6732	0.5504	1.5349
mAb104-DX8951	0.4338	0.4273	1.1563
	0.5654	0.4110	1.1890

Supplementary Figure . Uncropped blots and densitometry analysis for **Figure S5 (B)** for pH2AX, pAKT, pERK1/2, GAPDH, HER2, AKT, ERK1/2 from NCI-H1650 week 3 tumor samples.

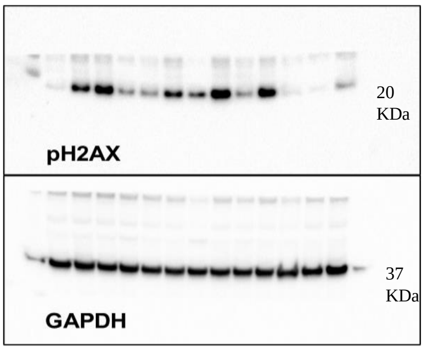

	pH2AX/GAPDH
IgG1	0.0370
	0.4824
	0.9322
104	0.2492
	0.2733
Tras	0.6131
	0.3967
TDM1	1.2001
	0.2611
IgG1-DX8951	0.9395
	0.0402
mAb104-DX8951	0.0164
	0.1167

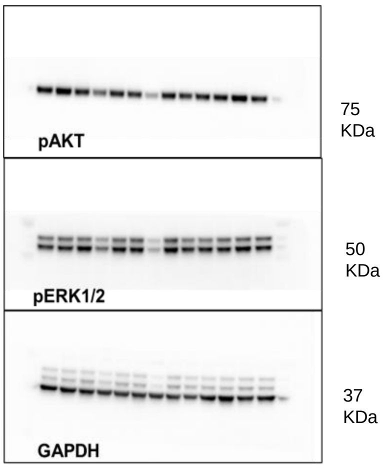

	pAKT/GAPDH	pERK/GAPDH
IgG1	1.0890	1.4447
	1.4107	1.5407
	1.3244	2.0086
104	0.8874	1.0666
	1.4808	1.8816
Tras	1.4521	2.0397
	0.5541	0.6172
TDM1	1.7019	2.3997
	1.6253	2.1353
IgG1-DX8951	1.2777	1.5497
	1.0731	1.3352
mAb104-DX8951	1.8521	2.1317
	1.1524	1.7461

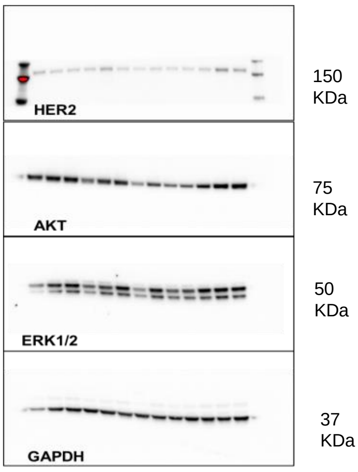

	HER2/GAPDH	AKT/GAPDH	ERK/GAPDH
IgG1	0.8212	4.0054	2.2105
	0.2416	2.0341	2.4146
	0.1754	1.6526	2.6141
104	0.1924	0.9466	1.4398
	0.3716	1.4953	2.4319
Tras	0.2031	1.6364	3.1299
	0.1270	0.6297	1.2678
TDM1	0.1701	1.1062	2.9535
	0.1627	0.7839	2.2152
IgG1-DX8951	0.1421	0.7391	1.8127
	0.2135	1.2298	2.4852
mAb104-DX8951	0.5440	1.9059	3.0732
	0.3577	1.6857	2.3678
